# Influence of Dietary Heritage in a Restricted Geographic Area and Role of Food Additives on Risk of Recurrent Kidney Stone

**DOI:** 10.3390/nu16172984

**Published:** 2024-09-04

**Authors:** Piergiorgio Bolasco, Giorgio Reggiardo

**Affiliations:** 1 Nephrolithiasis and Rare Nephrocalcinosis Study Group, Italian Society of Nephrology, 00185 Rome, Italy; 2 Department of Biostatistics, Consortium for Biological and Pharmacological Evaluations (CVBF), 27100 Pavia, Italy; giorgio.reggiardo@gmail.com

**Keywords:** nutrition, nephrolithiasis, Kidney Stone Formers, diet recall, urinary stone risk, food additives

## Abstract

Dietary factors may be implicated in the formation of kidney stones and should be closely monitored. To achieve this aim, patients are routinely assessed by means of generic dietary recall, a tool widely used by authors in a range of extensive patient populations to record food intake; the findings obtained, however, may be skewed due to dietary variations and underestimation of the effect of food additives. Fifty Frequent Kidney Stone Formers (FKSFs, mean age: 54.3 ± 13.9 years) with normal kidney function, absence of comorbidities, and reliable compliance were selected from a total of 68 patients’ resident in Sardinia, an Italian island where genetic admixtures have been relatively rare for generations. The study, conducted from 1 January 2020 to 31 December 2023, was aimed at assessing nutritional values based on the meticulous recording of food quantities, quality, and potential modifications related to food preparation. Patients were selected during an initial clinical check-up and all efforts made to ensure they were capable of reliably recording all food and drinks consumed. A seven-day food diary was provided in which food and drink intake and their impact on 24 h urine output was recorded. The following parameters were measured in both foods and urine output: citrates, oxalates, calcium, phosphorous, uric acid, proteins and nitrogen compounds, magnesium, sulfates, potassium, carbohydrates, free fatty acids. Study outcomes established the presence of hypocitraturia, hyperoxaluria, hypercalciuria, and moderately high levels of nitrogen compounds. Univariate analysis followed by multivariate analysis for further confirmation were performed and the following observations made. Citrate intake correlated with citraturia but did not promote oxaluria; calcium intake promoted onset of sulfaturia, azoturia, and ammoniuria, whilst magnesium correlated with magnesiuria but not with oxaluria, calciuria, phosphaturia, and azoturia; sulfate intake elicited onset of azoturia but not kaliuresis; potassium intake promoted oxaluria and protein intake resulted in onset of ammoniuria and azoturia. (A) The chemical composition of urine based on dietary intake is hard to predict without taking into account the presence of dietary and urinary interferents; (B) the geographic isolation of patients studied underlines the importance of epigenetics in maintaining a traditional dietary heritage. (C) Moreover, the widespread use of food additives should consistently be taken into account to ensure a correct diagnosis of FKSF and set up a valid treatment plan.

## 1. Introduction

Despite the extensive diffusion of nephrolithiasis worldwide, a limited number of epidemiological studies have been conducted, and they have yielded no reliable rates of incidence or prevalence, largely due to a failure to record cases referred to primary care or hospital services, with availability of only a limited number of hospital discharge records. Seven southeastern European countries have reported prevalence rates ranging between 3.4% and 6.8%. The most recent Italian reports confirm a prevalence of renal lithiasis ranging from 6.8% to 10.1%, with relapses in the range of 30% to 50% within 5 to 10 years of the initial event, predominantly amongst males [1,2,3]. A prevalence rate of 8.8% has been reported in the US, with a similar male prevalence [4]. Literature reports have demonstrated numerous correlations in both industrialized and rural settings, linking renal lithiasis to lifestyle and dietary habits in patients affected by kidney stones, particularly Frequent Kidney Stone Formers (FKSFs). Several reports, moreover, indicate how causal factors may bridge the gap between epigenetic causes, dietary factors [5,6], and environmental [7] and climate-related aspects [8,9]. In areas partially or fully isolated either geographically and/or due to ethnicity, the incidence and prevalence of frequent kidney stones (FKS) affect up to 15% of the population. These geographic/ethnic limitations are mainly represented by living in a condition of insularity and/or significant differences in eating habits due to social, dietary, or religious heritage and readily disposable income amongst FKSFs, even in a metropolitan setting. Under insular conditions, epigenetic or Mendelian inheritance patterns capable of affecting dietary heritage amongst first- to third-degree kinsmen, as stated at interview by subjects affected by FKS, should be regarded as a potential predisposing factor in the formation and relapse of kidney stones [10,11,12,13,14]. Sporadic studies investigating the incidence of kidney stones in an insular setting have referred to the effect produced on study outcome by both climate factors and influence exerted by urbanization [15,16,17]. Sardinia, a major island in the central Mediterranean situated far from the coasts of the Italian peninsula and characterized, particularly in the south of the island, by sub-tropical temperatures, has been subjected to limited admixtures with other Italian or Corsican populations, thus representing, for epigenetic purposes, an ideal epidemiological “observatory” featuring a well-established genomic imprinting for numerous diseases, including type 1 juvenile diabetes, multiple sclerosis, ß-thalassemia glucose-6-phosphate dehydrogenase deficiency, and celiac disease [18,19,20]. Dietary habits on the island are strongly influenced by agriculture and sheep farming in the hinterland and the fishing industry in coastal areas, with a cuisine rich in sodium salts, protein, carbohydrates, foods rich in oxalates, calcium, and phosphates, likely genetically associated with non-protective low citrate levels. Although currently lacking, a study investigating the presence of nephrolithiasis on the island might provide food for thought with regard to the formation and relapse of kidney stones, which would likely prove to be at variance with data collected in other Mediterranean countries or regions [20,21]. The present study was conducted retrospectively from 1 January 2020 through 31 December 2023 with the aim of highlighting the association between kidney stone formation and current eating habits, as meticulously recorded by fully compliant FKSF patients prior to initial nephrological assessment. The accurate form of recording requested differed significantly versus literature findings based on generic dietary assessments in FKSF patients, which failed to take into account the frequent dietary variations which may occur both during and between meals, and may not necessarily be communicated to the interviewer [22,23]. The primary aim of this study was to evaluate the meticulously recorded dietary recall to extract details relating to the quantity and quality of foods consumed, taking into account any variations foods may have been subjected to either when consumed individually or as an added ingredient in more complex recipes. This process allowed us to calculate the consumed intake of proteins, calcium, phosphorus, oxalates, citrates, sulfates, magnesium, potassium, uric acid, carbohydrates, and free fatty acids and compare these with detected urinary levels of citrates, oxalates, potassium, calcium and phosphorus, uric acid, sulfates, magnesium, urea, and ammonium, and thus determine the presence of correlations between the above pro- or anti-lithogenic food components and concentrations of the same in 24 h urines. Lastly, we aimed to emphasize potential dietary and urinary influences/interferences produced by the presence of additives in the foods consumed. These evaluations may contribute to the optimization of therapeutic or nutritional interventions.

## 2. Materials and Methods

Patients taking part in the study were asked to meticulously record all foods and drinks consumed from breakfast onwards, to include main meals, snacks or any other additional items. The main foods consumed included various types of bread and pizza, fresh or dried pasta, meat and cured meats, fish, seafood, crustaceans, vegetables, pulses, eggs, all types of both sugary and sugar-free drinks, including freshly squeezed citrus juices, all types of alcoholic beverages and milk and milk-based products, confectionery, chocolate, butter, cheeses and yogurt, all types of fruit and fruit juices. Patients were asked to weigh all foods prior to consumption or use as an ingredient in a recipe; they were, moreover, required to specify how the product was consumed—either raw or cooked by boiling, steaming, microwave cooking, or roasting to allow element losses to be assessed, and to mention the addition of a range of flavor-enhancing seasonings. Dietary recall was recorded daily from Monday (breakfast) through Sunday (evening meal) over the week prior to 24 h urine collection to be used in determining the metabolic profile of various urinary constituents. The nutritionist and/or nephrologist were in continuous contact with their patients, with all investigations and dietary recalls taking place within 36 months of study commencement. All foods consumed, cooking methods, and weights were recorded in an Excel spreadsheet containing seven columns, one for each day of the week to allow calculation of an exact daily mean for each type of food or drink. Calculations thus obtained were subsequently normalized based on the cooking method applied. Patients were asked to refrain from taking any drugs or supplements that might affect the results of metabolic screening of urines. Between 1 January 2020 and 31 December 2023, sixty-eight FKSF patients were referred to our clinic for initial nephrological assessment. 

Exclusion criteria: Chronic Kidney Failure with estimated Glomerular Filtration Rate (eGFR) < 70 mL/min normalized for body surface area and age, primary or secondary hyperparathyroidism, myeloproliferative disorders, primary or secondary renal tubular disorders, inflammatory bowel diseases, BMI < 18.5 or signs of malabsorption and/or malnutrition, metastatic tumors, acute, subacute, or chronic pulmonary disease, any form of gastrointestinal surgical by-pass, recent or ongoing primary gout, administration of drugs in the treatment of uric acid dysmetabolism, as well as citrates, acetazolamide, hydrochlorothiazide, beta-lactam antibiotics, diuretics and routine use of alkalizing substances and/or vitamin C, recent or routine use of indomethacin, losartan, probenecid, salicylic acid, pyrazinamide, and routine administration or high doses of vitamin D. Patients were also excluded if, prior to or during clinical assessment, they were deemed unreliable or incapable of meticulously recording dietary intake. Based on the above, a total of 50 patients were recruited to the study (27 females (54%) and 23 males (46%)—mean age 54.3 ± 13.9) [24,25]. To obtain an accurate metabolic reading, urines collected over a 24 h period were divided equally into two containers, the first pretreated with hydrochloric acid and the second chlorhexidine, and forwarded to a well-established certified lab (Kidney Stone Metabolic Profile Service by LIT—Turin, Italy) for testing for urinary constituents: oxalates, inorganic calcium and phosphorus, magnesium, citrates, sulfates, sodium, potassium, chloride, urates, ammonium, urea, pH, and creatinine [24,25]. Dietary recall allowed data relating to total proteins, carbohydrates, and free fatty acids to be acquired. During dietary recall week, a sample of venous blood was obtained from each patient for measurement of intact parathyroid Hormone (PTHi )pg/L, sodium and potassium in mEq/L, calcemia and phosphoremia in mg/dL, uricemia in mg/dL, and estimation of GFR using the new CKD-EPI race-free formula [26]. Protein intake, calculated based on urea nitrogen appearance (UNA) as described in Mitch et al. [27], was combined with urea nitrogen present in 24 h urines, nitrogen deriving from 24 h urine creatininuria and ammoniuria + non-urinary fecal nitrogen output. To calculate total protein intake (g/kg) from daily dietary recall, increased nitrogen compounds available for distribution throughout body water in the absence of kidney failure in patients in a metabolic steady state were assumed to be zero. Blood gas analysis was not deemed necessary due to a lack in our patients of severe renal and/or pulmonary insufficiency capable of impacting blood acidosis or bicarbonatemia. Due to the difficulty of calculating the addition of salt to foods consumed, sodium chloride intake was not quantified. Daily sodium intake was extrapolated and calculated based on 24 h natriuresis [28]. Nutritional content of all food constituents, including carbohydrates and free fatty acids, was extracted from a series of web-based data portals: CREA (Italian Council for Agricultural Research and Assessment of Agrarian Research) [29,30,31], BDA (Database of food composition for Epidemiological Studies in Italy) [32,33,34,35,36,37,38], and the USDA (U.S. Department of Agriculture) platform [39,40,41]. Other web portals were used to provide an accurate calculation of citric acid present in fruits and vegetables [42] as well as quantities of uric acid contained in purine-rich foods [24]. To aid understanding of the results obtained, Table 1 lists the most recently established normal reference ranges for the main urinary components [43,44,45,46,47], although it should be taken into account that these parameters are subject to wide variations according to the author consulted and numerous variables. 

## 3. Statistical Analysis

Descriptive statistics, including mean and standard deviations (SDs) for continuous variables, were used. All variables were tested for normality using the Kolmogorov–Smirnov test. The relationship between two continuous variables was assessed by means of Pearson’s correlation coefficient, with the coefficient being calculated for each comparison independently. Linear regression models were used to analyze the linear relationship between two variables and the results presented with β coefficients and corresponding 95% confidence intervals (CIs). Regression lines were drawn by simple regression analysis using SPSS software. A multivariate regression model was used to simultaneously capture the effect of multiple dependent variables by more covariates. No replacement method was used for missing data. Statistical tests were two-sided and a value of *p* ≤ 0.05 was considered as statistically significant. All analyses were performed using Statistical Package for Social Science (IBM SPSS Statistics for Windows, Version 29.0, IBM Corp., Armonk, NY, USA).

## 4. Results

**Figure 1:**
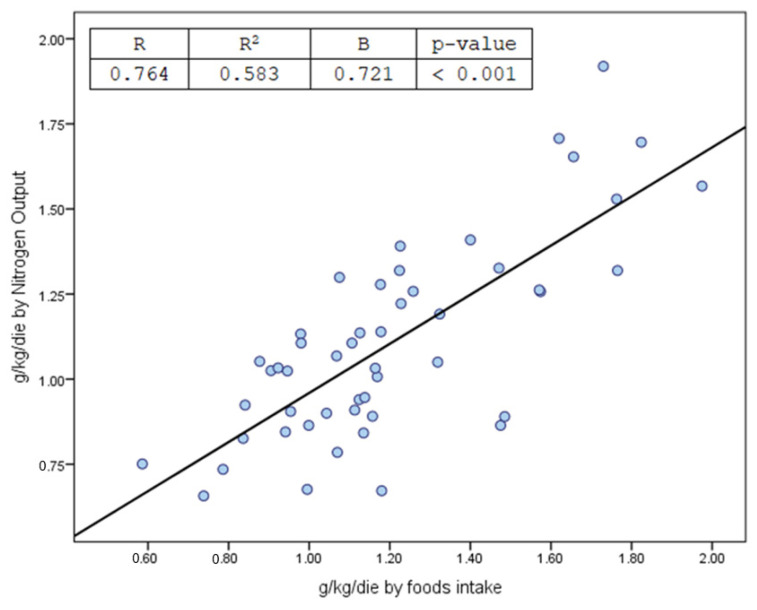
Correlation between protein intake from recall food records and calculated from urinary and non-urinary nitrogen compounds [27].

In the fifty FKSF patients recruited to the study, causal diagnosis based on 24 h urine collection was lithiasis caused prevalently by hyperoxaluria (twelve patients), hypercalciuria and hyperoxaluria (ten patients), hyperuricemia (five patients), calcium phosphate (nine patients), hypercalciuria and hyperuricemia (seven patients), hyperuricemia and hyperoxaluria (two patients), isolated hypocitraturia with no other abnormalities (five patients). Unfortunately, only 11/50 patients (22%) recovered their stones to perform infrared spectrometry analysis (calcium phosphate (four patients), uric acid (four patients), calcium oxalate (three patients)), so it was not possible to make any correlation between food components and urinary elements. Table 2 reports patients’ personal data, serological parameters, body parameters, and renal function status, in addition to the findings of a series of linear correlation analyses performed on patients’ plasma and urine to assess levels of sodium, potassium, calcium, phosphorous, and uric acid, whilst Table 3 provides values obtained for the above-cited elements in foods consumed on a daily basis and their urinary output. Moreover, Table 3 highlights patients who displayed values outside the reference range for citraturia, oxaluria, calciuria, phosphaturia, azoturia, sulfaturia, magnesiuria, and ammoniuria; urinary nitrogen levels > 12 g/24 h were deemed high, corresponding to a protein intake of between 1.2 and 1.4 g/kg/day, as calculated from total protein counts recorded for foods consumed daily. Accordingly, Figure 1 illustrates Pearson’s correlation coefficient (g/kg/day) obtained for total proteins deriving from a daily mean of recorded foods and urinary proteins based on the presence of nitrogen compounds and fecal nitrogen output from 24 h urine collection [27], which revealed a markedly significant correlation between proteins deriving from recorded food intake and total urinary nitrogen × 6.25. Other noteworthy findings include marked hypocitraturia/24 h corresponding to one-third of anti-lithogenic values and less than one-tenth of citrates introduced with food, as well as calciuria of almost twice the normal reference values and 46% less than dietary intake. All other elements tested fell within normal reference range; however, compared to dietary intake, urinary phosphates were 51% lower, urinary sulfates 49% lower, urinary magnesium 73% lower, whilst urinary potassium and uric acid corresponded to dietary intake, and urea, ammonium, and creatinine derived from a dietary protein intake of 1.15 g/kg/day. Lastly, daily intake of carbohydrates and fatty acids fell moderately outside recommended limits [48,49]. Table 4 provides details of univariate correlation analysis performed to ascertain conformity between each food constituent and urinary equivalent, taking into account the impact of potential confounding factors such as intake of foods rich in carbohydrates and saturated fatty acids. Several significant correlations were observed between citrate intake and calciuria, calcium intake and urinary nitrogen, phosphate intake and kaliuresis and urinary nitrogen, uric acid intake and urinary nitrogen, protein intake and urinary nitrogen, sulfate intake and urinary nitrogen. However, to verify the univariate analysis relationship data between food constituents and urinary excretion of the same, multivariate regression analysis was performed. Table 5 highlights statistically significant associations based on a multivariate regression model comprising all elements analyzed. Citrate intake showed an inverse correlation with oxaluria, calcium intake correlated positively with sulfaturia, uricosuria, azoturia, and ammoniuria, dietary intake of magnesium correlated positively with oxaluria, but inversely with calciuria, phosphaturia, and azoturia, sulfate intake correlated positively with azoturia, but inversely with kaliuresis, potassium intake correlated positively with oxaluria, and protein intake correlated positively with azoturia and ammoniuria. Univariate–multivariate correlations were calculated based on the impact produced by dietary intake of carbohydrates and saturated fatty acids with all urinary components, yielding no significant correlations. Despite a variation in urinary pH from 5.4 to 7.2 in 24 h urine collection, at univariate and multivariate correlation analysis, no correlations were detected with any dietary elements, with the exception of a sole significant correlation with phosphaturia mg/24 h (*p* = 0.007). It was not possible to establish correct estimations of the actual sodium content of table salt used in cooking; sodium content was therefore based on 24 h natriuresis corresponding to a table salt intake of 6.4 ± 6.5 g/day [50,51]. Mean calorie intake in our study sample was 1896.3 ± 1307.9 Kcal/day. 

## 5. Discussion

### 5.1. Urinary Effects of Citrate Intake

As expected, the recurrent presence of fruit and/or vegetables and/or pulses in patients’ daily diets correlated with urinary citrate levels, although failing to reach anti-lithogenic concentrations in the patients studied, with 39/50 (78%) of FKSF displaying significantly low or modest levels of citraturia. It should also be taken into account that an isolated finding of hypocitraturia with no evident irregularities in other urinary constituents, previously detected in 14% of the 50 FKSFs, may alone facilitate kidney stone formation and relapse under conditions of urinary supersaturation (marked, although at times temporary, dehydration and/or poor reintegration of liquids and/or high ambient temperatures) [52]. The cause of this marked reduction in urinary citrates was not linked to dietary factors, with no evidence of recruited patients recording marked imbalances in food consumption, such as high intake of meat or cured meats or plant products such as fruit, vegetables or pulses [53]. Epigenetic and familial factors, potentially characterized by increased intestinal reabsorption at the brush border of the small intestine, but directly impinging on the apical membrane of the proximal tubule responsible for modifying functionality of Na/dicarboxylate co-transporters (NaDC-1), could be taken into serious consideration as a causal factor [54,55]. The finding that 39 (70%) of 50 FKSF patients had first- and second-degree familiarity for the above is of particular interest. Indeed, familial genetic factors for this condition have been identified in pediatric populations from isolated geographic areas [56], as well as in adults from a series of isolated regions in Turkey, Portugal, Brazil, and Yucatan [57]. Another predictable event that correlated significantly with the intake of citrate-containing foods in the population studied was represented by a reduction in oxalates, as demonstrated by multivariate regression analysis [58,59]. Particularly, in the Sardinian region, the widespread production and consumption of citrus fruits underlines the relevance of recent findings relating to the effect of lemon juice on reducing episodes of relapse of calcium oxalate stones (CaOx) [60]. This association between citrates and decreased presence of urinary oxalates is not justifiable solely at the renal level, but likely implicates genomic alterations capable of eliciting increased citrate absorption by means of specific transporters from the intestine to the kidney expressed on enterocyte brush border membranes for class Na^+^/citrate transporters, specifically NaDC-1 and hNaDC-1 [61]. 

### 5.2. Urinary Effects of Calcium Intake

Dietary calcium intake recorded by patients fell within recommended daily limits [62]; multivariate regression analysis revealed a positive correlation between dietary calcium intake and sulfaturia, uricosuria, azoturia, and ammoniuria. The correlation between dietary calcium and nitrogen deriving from proteins of animal origin is well known due to a high presence of calcium in numerous foods, particularly proteins of animal origin. The findings obtained in this study are in line with the dietary traditions observed in this island population, with the cuisine of hinterland areas typically favoring high-protein and calcium-containing foods, such as poultry, milk, dairy products (particularly mature sheep’s cheese), cabbage, beans, lentils, chick-peas, pork (particularly traditional suckling pig), and lamb. Coastal populations, however, tend to consume a fish-based diet, including freshly caught and simply cooked tuna and swordfish and high consumption of locally canned oily fish, prawns, and crustaceans, as well as mussels and clams, either freshly cooked or preserved in oil or brine [51,63,64,65,66]. The correlation between calcium and sulfur is of particular interest due to the wide use of calcium sulfate as an additive in the form of sulfuric acid and sodium sulfate and bisulfate (E514 I and II), potassium sulfate (515 I and II) and calcium sulfite (E226), but mainly calcium sulfate (E516), the use of which has been approved by the FAO and FDA. Calcium sulfate is used extensively as an additive across all food categories, including in milk, dairy products and condensed milk, numerous types of cheeses, confectionery, baked goods, pasta, condiments, nutritional supplements, breakfast cereals, rice, and yeast for the fermentation of beer; the compound is also used as a firming agent [67], raising agent, and to boost food sapidity. The non-hazardous nature of calcium sulfate has recently been reassessed and its use extended to numerous foods; for this reason, it was not deemed necessary to quantify and to regulate acceptable daily intake (*quantum satis*). Indicatively, dietary introduction of calcium sulfate is in the range of 5–11 mg/kg/day [68], with an average consumption that could be assumed in the population of 50 patients recruited of at least >500 mg/day; furthermore, calcium sulfite and bisulfite are also widely used as additives (E226–227) to preserve and extend organoleptic qualities in fruit juices, canned fruit, jams and marmalades, cured meats, dried fruits and, particularly, wines at regulated amounts ranging from 200 to 400 mg/L, used exclusively for their anti-oxidant and antiseptic properties. The quantities used of these additives justifies the positive correlation detected at multivariate analysis between dietary calcium and sulfaturia/24 h. Accordingly, sulfate intake in our patients corresponded to 682 mg/day, approximately 10–20% of which was composed of calcium sulfates and bisulfites. Renal epithelial cells produce ammonia, largely in the proximal tubule, with renal excretion influenced by protein intake, particularly glutamine, likely the primary substrate underlying its presence in urine [69]. Several commonly consumed foods featuring a high calcium and protein content correlate significantly with urinary excretion of nitrogen compounds, including urea and ammonium, although in our analysis, they failed to correlate with uric acid [29,34,35]; these foods include all meat products and cured meats, with 20–30 g protein and 10–60 mg calcium per 100 g, shellfish, fresh or canned oily fish, and crustaceans, with 11–25 g protein and 90–220 mg calcium per 100 g, all cheeses, from soft to mature, with 12–30 g protein and 25–1150 mg calcium per 100 g [70,71]. Common additives that also impact urinary excretion include ammonium or calcium polyphosphate (E544 and E545), widely used in the production of meat products, cured meats, and cheeses as an emulsifier and stabilizer, in cocoa-based products, or as raising agents in the commercial food industry in Italy and the US [72]. Ammonium carbonate (E503) and/or ammonium hydrogen carbonate (E503ii), obtained by sublimating a mixture of ammonium sulfate and calcium carbonate, are present at significant quantities throughout a wide range of breakfast products, including biscuits and snacks marketed by the European and US food industries and also employed, at times, in home-baked biscuits [73,74].

### 5.3. Urinary Effects of Magnesium Intake

Magnesium is found across all types of foods, being the second most abundant cation in vertebrates. In patients studied, dietary intake fell within the recommended levels of 320–420 mg/day and correlated positively with magnesiuria, which slightly exceeded the normal reference range. Our analysis confirmed the importance of magnesium intake in reducing levels of oxalates, calcium, phosphate, and azoturia, a finding substantiated by the correlation revealed at multivariate analysis between dietary intake and decrease in oxaluria. Indeed, oxalate reduction is initiated at the intestinal level, with both calcium and magnesium binding to oxalates, resulting in an increased fecal excretion and consequent decrease at the renal level [75,76]. Multivariate regression analysis confirmed a negative correlation for the effect of magnesium on renal excretion of phosphates, thus contributing to the elimination of approx. 80% of calcium present at the intestinal level and to increasing reabsorption of calcium and phosphorous in the ascending segment of the proximal tubule [77]. Dietary magnesium directly inhibits expression of sodium-dependent protein co-transporters NaPi-IIa and NaPi-IIc by renal receptors, and indirectly by inhibiting PTH secretion, thus reducing its stimulant effect on the above-cited sodium-dependent co-transporters [78,79]. Table 5 shows a further negative correlation between intake of magnesium-containing foods and the presence of urinary urea nitrogen and urinary ammonia. It is likely that the presence of magnesium in all foods of animal and plant origin consumed by our patients might have resulted in a renal imbalance between magnesium intake and azoturia. Indeed, from the list of foods weighed and recorded on a daily basis, 12% of patients were found to consume mainly animal and plant proteins, whilst 60% opted for magnesium-rich plant foods such as salads or pulses. Therefore, in the absence of reports on the correlation kinetics manifested between magnesium and proteins in transportation and reabsorption at an intestinal and renal level, it can be assumed that this effect was likely caused by the dietary habits of the patients studied.

### 5.4. Urinary Effects of Sulfur Intake

The correlation between dietary intake of animal proteins and sulfates is well-known. A factor of 1/6.25 is applied to convert dietary protein (g) to nitrogen (g) and 1/18.9 to convert nitrogen (g) to sulfur (g); moreover, allowing for some degree of variability, a ratio between protein intake of 70–75 g/day and an 85% reduction of sulfaturia (g) is generally acknowledged. In our patient sample, this ratio rose to approximately 88%. Generally, a diet rich in animal proteins elicits excretion of higher amounts of sulfates in 24 h urines, confirmed in our patient sample by the finding of a positive correlation at multivariate analysis between protein intake and sulfaturia. The conversion factor in urines, however, differs, with inorganic sulfur (g/d) being calculated on the difference between total urinary sulfur (g/day) and urinary nitrogen (g/day)/18.5 [80]. Sulfates are excreted mainly by the kidneys and may be prone to wide variations at the intestinal level due to fecal sulfur losses [79,81,82,83,84]. Natural dietary intake of sulfates should also take into account the numerous sulfur-containing additives used across a wide range of protein food products, including sodium sulfate and bisulfate (E514 I and II), potassium sulfate (515 I and II), calcium sulfate (E516), sodium sulfite (E223) calcium sulfite (E226), sodium metabisulfite (E223), and potassium bisulfite (E228), the use of which has been approved by the FAO and FDA (this list is in Annex II of Regulation (EC) No 1333/2008) [72]. In our patient sample, the amount of sulfates was clearly correlated with urinary urea deriving from a protein intake of 1.2 g/kg/day, composed largely of animal proteins, with considerably lower intake of plant-based products, as confirmed by a positive correlation at multivariate analysis between sulfate/protein/azoturia intake, and a significant negative correlation between sulfate intake and urinary potassium concentrations. Our patients’ eating habits tended towards a lower dietary intake of potassium, which was almost completely reabsorbed in the proximal convoluted renal tubule and the ascending Henle loop (Table 3), thus maintaining physiological plasma levels of potassium (Table 2). Furthermore, a high sodium intake would likely result in dysregulation of potassium transporter mechanisms [85] and reduced secretions along the distal convoluted tubule. 

### 5.5. Urinary Effects of Potassium Intake

In our patients, however, this positive correlation between potassium and oxaluria may be due not only to the choice of eating habits and preference for plant-based foods containing moderate amounts of potassium and oxalates. Indeed, dietary recalls completed by the 50 patients studied tended to point towards two distinct eating patterns. The first, a more common and widespread pattern, included the consumption of foods with an average oxalate content (25–50 mg/100 g), ranging from potatoes cooked in various ways, particularly fried (100 mg/100 g), various types of beans cooked in a variety of ways, whilst the second was more typical of the local cuisine based on the consumption of raw or cooked fava beans, abundant use of tomato salsa throughout the year, fresh figs and prickly pears. A characteristic eating habit common to the hinterland areas of the island relates to an extensive use of high-oxalate-content vegetables (650–750 mg/100 g), including raw or cooked chard and cooked wild spinach foraged by the hinterland populations. As illustrated previously, in the population studied, dietary intake of potassium, on reaching the kidney, is almost wholly reabsorbed along the proximal convoluted tubule and ascending Henle loop. Thus, the reported correlation between a diet rich in potassium-containing plant foods and increased risk represented by high levels of oxalates is indisputable [86,87]. 

### 5.6. Urinary Effects of Protein Intake

It is a well-established fact that protein intake results in an increased excretion of urea and ammonium [51,69,70,88]. In the patient cohort studied, multivariate analysis failed to confirm a positive correlation with uric acid, with only two patients (4%) exceeding the urinary uric acid threshold of 600–650 mg/day. As mentioned previously, our patients were not affected by any acute or chronic diseases that might have altered metabolic steady state, resulting in a protein/nitrate-induced equilibrium. Thus, calculation of the total amount of nitrogen compounds detected in urine facilitates the estimation of mean protein intake in FKSF patients at the time of their first metabolic screening, confirmed by the correlation shown in Figure 1, showing how total urinary nitrogen × 6.25/kg weight of patients studied displayed a significant correlation with protein intake in g/kg/day, as calculated from patients’ food diaries.

## 6. Conclusions

The small number of patients studied represents one of the main limitations of the present study. This paucity of patients was due partly to the need to continuously monitor each individual patient to ensure the correctness and reliability of data relating to the recording of all foods on the dietary recall, an onerous task that cannot be performed in routine clinical practice. The several significant correlations obtained at univariate analysis vanished using multivariate analysis due to the effect exerted by other inference elements on each individual food constituent. The conclusions obtained in this study indicate a series of important observations to be taken into account during screening of FKSF patients. (a) An insular epigenetic inheritance recycling enhances understanding of the way in which incidence and prevalence of familial and genetic imprinting tends to produce a more significant effect in geographically isolated regions; (b) this significant geographic isolation and determination to maintain and pass down an ancient dietary heritage contribute to the conservation of a series of dietary habits linked to the preparation and serving of a traditional cuisine; (c) the largely underestimated, but significant use of additives, duly approved and classified as non-hazardous by European legislation, represents a novel factor to be taken into account going forwards due to their ability to derange the prescription of treatment and personalized nutrition plans. The findings obtained in our limited study should be further confirmed in accurately conducted large-scale dietary studies in FKSF patients aimed at investigating the influence exerted by epigenetic, environmental, and geographic factors and cultural traditions. These studies should, moreover, be extended to examine the issue of analytical interferences in urine resulting from the widespread use of food additives. 

## Figures and Tables

**Table 1:** Reference value ranges in 24 h urine collection.

Citrates: ≤320 mg/day for females and <360 mg/day for males
Oxalate: <40 mg/day
Uric acid: <600 mg/day for females and <650 mg/day for males
Calcium: <220 mg/day for females and <250 mg/day for males
Inorganic phosphate: <1085 mg/day
Ammonium (NH_3_): <50 mmoL/day
Sulfate: 960–2900 mg/day
Magnesium: <75 mg/day
Natrium: 50–200 mEq/day
Kalium: 50–100 mEq/day
Nitrogen: 12–24 g/day

**Table 2:** Plasmatic and urinary parameter descriptive and linear regression analysis.

	Mean ± SD		
N. Patients	50		
male/females	27/23		
age, years	54.3 ± 13.9		
Usual average diuresis pre-screening, mL/24 h	1232 ± 633		
Collected urine, for metabolic tests, mL/24 h	2401 ± 968		
Weight, kgs	70.0 ± 13.8		
Body Surface, m^2^	1.78 ± 0.19		
B.M.I., Kg (height × height) m^2^	20.8 ± 3.7		
Patients with Hypertension, number and %	7 (14)		
Diabetic patients, Numbers and %	2		
eGFR by CKD-EPI, mL/min/body surface, m^2^	97.9 ± 16.7		
plasmatic Sodium, mEq/L	140.5 ± 2.9		
plasmatic Potassium, mEq/L	4.3 ± 0.3		
plasmatic Calcium, mg/dL	9.5 ± 0.6		
plasmatic Phosphorum, mg/dL	3.3 ± 0.5		
plasmatic Uric Acid, mg/dL	5.5 ± 1.6		
PTH, ρg/mL	54.9 ± 5.5		
Linear Regression between plasmatic and urinary values	R	R^2^	*p*
Plasmatic Sodium vs. Sodiuria	0.047	0.02	0.766
Plasmatic Potassium vs. Kaliuria	0.220	0.05	0.152
Plasmatic Calcium vs. Calciuria	0.334	0.11	0.022
Plasmatic Phosphorum vs. Phosphaturia	0.430	0.02	0.343
Plasmatic Uric Acid vs. Uricuria	0.104.	0.01	0.497

**Table 3:** Daily food element intake and corresponding urinary values. Number and percentage of patients with outsider urinary concentration levels.

Content in Foods Consumed Daily		Urinary Parameters, 24 h		Outsiders (n, %)
Daily Intake Citrate, mg	1414.8 ± 635.6	Citrate, mg	105.5 ± 1.52	<43 (86%)
Daily Intake Oxalate, mg	200.8 ± 130.4	Oxalate, mg	163.9 ± 103.3	<12 (24%)
Daily Intake Calcium, mg	935.2 ± 396.2	Calcium, mg	505.3 ± 192.6	>23 (46%)
Daily Intake Phosphate, mg	1337.8 ± 1452.2	Phosphate, mg	657.3 ± 267.6	<1 (2%)
Daily Intake Proteins, g/kg/day	1.2 ± 0.3	Nitrogen, g	9.8 ± 2.3	>6 (12%)
		Ammonium, mmol	28.5 ± 4.5	<50 (100%)
Daily Intake Sulfur, mg	682.7 ± 240.9	Sulfate, mg	349.9 ± 153.6	<50 (100%)
Daily Intake Magnesium, mg	353.2 ± 58.0	Magnesium, mg	93.0 ± 33.8	>30 (60%)
Daily Intake Potassium, mEq/L	56.2 ± 21.0	Potassium, mEq/L	54.2 ± 21.2	-
Daily Intake Uric Acid, mg	476.9 ± 149,1	Uric Acid, mg	505.3.6 ± 267.2	>2 (4%)
Daily Intake Carbohydrates, g	316.0 ± 141.8	Carbohydrates, g	-	-
Daily Fat Free Acid, g	23.1 ± 11.2	pH	6.3 ± 0.7	-

< low values; > high values compared to reference normal range values.

**Table 4:** Correlation between oral intake daily elements and their urinary equivalents.

Elements	Spearman’s Rho Significance	Daily Intake Citrate, mg	Daily Intake Oxalate, mg	Daily Intake Calcium, mg	Daily Intake Phosphate, mg	Daily Intake Uric acid, mg	Daily Intake Proteins, g/kg	Daily Intake Magnesium mg	Daily Intake Sulfate, mg	Daily Intake Potassium mEq/L	Daily Intake Carbohydrates g	Daily Intake Saturated Fatty Acid, g
Urinary Citrate, mg/24 h	*Corr. Coefficient* *Significance*	0.326 0.021	0.047 0.728	0.070 0.628	0.198 0.168	0.173 0.230	−0.152 0.291	0.015 0.918	−0.067 0.734	0.113 0.435	−0.058 0.687	−0.133 0.139
Urinary Oxalate, mg/24 h	*Corr. Coefficient* *Significance*	0.079 0.586	0.210 0.229	−0.104 0.471	0.099 0.492	0.143 0.322	−0.058 0.687	−0.061 0.673	0.017 0.909	0.212. 0.140	0.027 0.851	0.010 0.949
Urinary Potassium, mg/24 h	*Corr. Coefficient* *Significance*	0.217 0.131	0.110 0.445	0.130 0.368	0.327 0.020	0.271 0.057	0.095 0.510	0.055 0.706	−0.151 0.297	0.190 0.187	−0.047 0.747	−0.121 0.403
Urinary Calcium, mg/24 h	*Corr. Coefficient* *Significance*	0.298 0.036	0.126 0.384	0.100 0.491	0.220 0.125	0.120 0.408	0.187 0.193	0.101 0.484	0.160 0.266	0.353 0.012	0.123 0.393	0.123 0.393
Urinary Phosphate, mg/24 h	*Corr. Coefficient* *Significance*	0.180 0.211	0.103 0.476	−0.270 0.853	0.185 0.200	0.219 0.126	−0.081 0.578	−0.051 0.723	−0.070 0.627	0.228 0.111	0.060 0.681	−0.163 0.258
Urinary Uric Acid, mg/24 h	*Corr. Coefficient* *Significance*	−0003 0.985	0.090 0.533	0.138 0.339	0.227 0.112	0.059 0.683	0.002 0.998	0.040 0.781	0.063 0.665	−0.440 0.761	0.054 0.71	−0.070 0.631
Urinary Nitrogen, mg/24 h	*Corr. Coefficient* *Significance*	0.093 0.519	0.132 0.362	0.324 0.022	0.334 0.018	0.308 0.030	0.425 0.002	0.143. 0.320	0.379 0.007	0.146 0.312	0.210 0.142	0.254 0.075
Urinary Sulfate, mg/24 h	*Corr. Coefficient* *Significance*	0.006 0.969	−0.138. 0.339	0.112 0.439	0.162 0.262	0.162 0.261	0.011 0.942	−0.014 0.924	−008 0.955	0.056 0.697	0.146 0.313	0.051 0.724
Urinary Magnesium, mg/24 h	*Corr. Coefficient* *Significance*	0.228 0.111	0.074 0.610	0.136 0.346	0.131 0.366	0.200 0.163	0.067 0.643	−0.039 0.789	−0.320 0.828	0.220 0.125	0.142 0.324	0.117 0.416

Corr. Coefficient: Correlation Coefficient by Spearman’s Analysis; Significance: p < 0.05.

**Table 5:** Multivariate regression analysis between the considered elements in food recalls and the corresponding ones detected in urine. Correlation coefficient and significance 2-tailed.

Source	Dependent Variable	B Coefficient	Type III Sum of Squares	F	Sig.
Intake Citrates 24 h (mg)	Citraturia 24 h, mg	24.54	386,552.249	4.523	0.040
	Oxaluria 24 h, mg	−2.64	17,135.339	9.544	0.004
Intake Calcium 24 h (mg)	Sulfaturia/24 h, mg	0.154	357,726.323	4.632	0.038
	Azoturia 24 h, mg	0.002	41.893	38.159	0.000
	Ammonuria, mmol/L	0.003	99.660	5.570	0.024
Intake Magnesium 24 h (mg)	Magnesuria 24 h, mg	0.025	5456.525	5.344	0.026
	Oxaluria 24 h, mg	−0.018	7776.369	4.331	0.044
	Calciuria 24 h, mg	−0.066	44,361.267	4.926	0.033
	Phosphaturia 24 h mg	−0.299	518,847.745	7.777	0.008
	Azoturia 24 h, mg	−0.101	5.289	4.817	0.035
Intake Sulfur 24 h (mg)	Azoturia 24 h, mg	0.005	5.208	4.744	0.036
	Kaliuria 24 h, mEq	−0.023	2286.043	5.693	0.022
Intake Potassium 24 h (mEq)	Oxaluria 24 h, mg	0.216	7812.135	4.351	0.044
Intake Protein	Azoturia 24 h, mg	3.985	140.094	127.607	0.000
	Ammonuria, mmol/L	3.800	129.865	7.258	0.011

Only values with a *p* < 0.05 are shown.

## Data Availability

The data presented in this study are available upon request from the corresponding author.
